# Multiple cutaneous ulcers revealing a primary cutaneous Epstein‐Barr virus‐positive diffuse large B‐cell lymphoma

**DOI:** 10.1002/ccr3.3239

**Published:** 2020-08-10

**Authors:** Billal Tedbirt, Sydney Dubois, Lucie Cellier, Priscille Carvalho, Aspasia Stamatoullas, Philippe Courville, Aurélie Deschamps‐Huvier, Pascaline Etancelin, Anne Deniel, Hervé Tilly, Fabrice Jardin, Pascal Joly, Vincent Camus

**Affiliations:** ^1^ Department of Dermatology Charles Nicolle University Hospital Rouen France; ^2^ Department of Hematology Centre Henri Becquerel Rouen France; ^3^ Department of Pathology Charles Nicolle University Hospital Rouen France; ^4^ Department of Genetic Oncology Centre Henri Becquerel Rouen France; ^5^ Department of Medical Oncology Centre Henri Becquerel Rouen France

**Keywords:** cutaneous lymphoma, cutaneous ulcers, Epstein‐Barr virus

## Abstract

Primary cutaneous EBV‐positive diffuse large B‐cell lymphoma is an exceptional and aggressive neoplasia with a poorer prognosis than other cutaneous lymphoma. Our observation points out the rarity of the presentation and the dismal clinical course.

## Graphical abstract

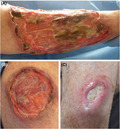

## 1 INTRODUCTION

Primary cutaneous Epstein‐Barr virus‐positive diffuse large B‐cell lymphoma (PC‐EBV‐DLBCL) is an exceptional and aggressive neoplasia with a poorer prognosis than other cutaneous lymphoma. This case highlights this immunosenescence‐associated lymphoid malignancy as an edifying cause of multiple skin ulcerations, distinct from other leg ulcers unrelated to arterial and venous disease.

Approximately, 20% of cutaneous lymphomas are B‐lymphocyte‐derived malignancies exclusively involving cutaneous site at the time of diagnosis. Cutaneous B‐cell lymphomas (CBCL) are classified into five distinct subgroups: primary cutaneous follicle center lymphoma (PCFCL); primary cutaneous marginal zone lymphoma (PCMZL); primary cutaneous diffuse large B‐cell lymphoma (PC‐DLBCL), leg type; intravascular diffuse large B‐cell lymphoma (IV‐DLBCL); and DLBCL, not otherwise specified (NOS). The prognosis of CBCL may vary depending on the subtype of CBCL. PCMZL and PCFCL are indolent forms, whereas PCLBCL, leg type, and IV‐DLBCL have an intermediate to aggressive clinical course.

The 2016 revision of the World Health Organization (WHO) classification of lymphoid malignancies recognized primary cutaneous Epstein‐Barr virus (EBV)‐positive DLBCL as a distinct entity within the group of “DLBCL NOS”.^1^ This is a rare but aggressive type of cutaneous lymphoma, which predominantly affects elderly and/or immunodeficient patients.^2^ However, this entity must be distinguished from EBV‐positive mucocutaneous ulcers (MCU) characterized by an isolated, circumscribed mucosal or cutaneous ulcers. By contrast, EBV‐MCU has an indolent course.^3^

We report a case of an elderly patient who presented multiple cutaneous ulcerations diagnosed as primary cutaneous EBV‐positive DLBCL.

## 2 CASE REPORT

**Figure 1:**
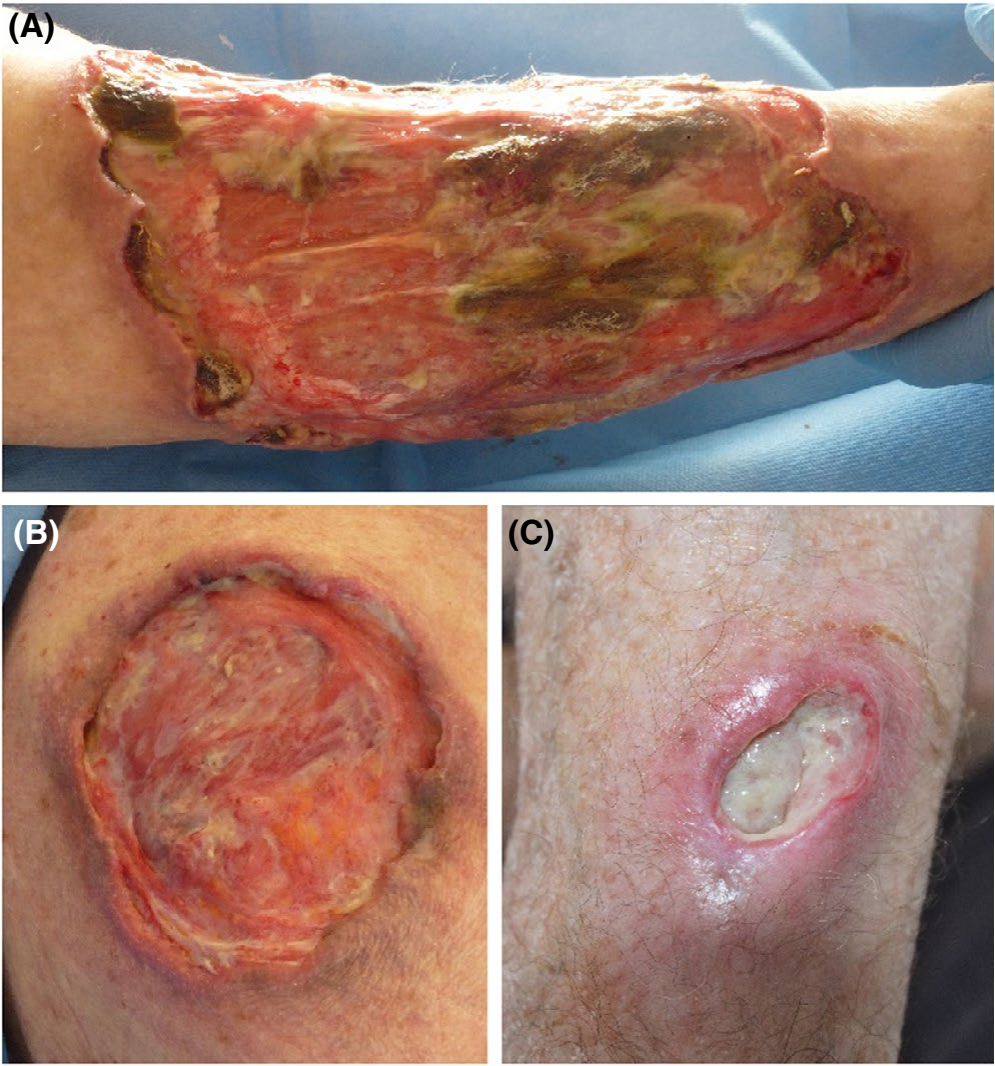
Large deep ulcers on the right lower leg (A), back (B) and left forearm (C)

A 77‐year‐old man presented with 4‐month history of unexplored diffuse nodules, which progressively evolved into painful large ulcers involving the tendons and which did not improve despite multiple oral antibiotics. He had a medical history of ischemic stroke, Parkinson's disease, and arterial hypertension. No previous biopsies or imaging studies was done in the four months before diagnosis. Clinical examination revealed impaired general condition and three large ulcerations measuring 15 × 10 cm on the lower limb, 8 × 6 cm on the back and 2 × 4 cm on the forearm (Figure 1A‐C). The patient had no mucosal lesions and no peripheral lymphadenopathies.

**Figure 2:**
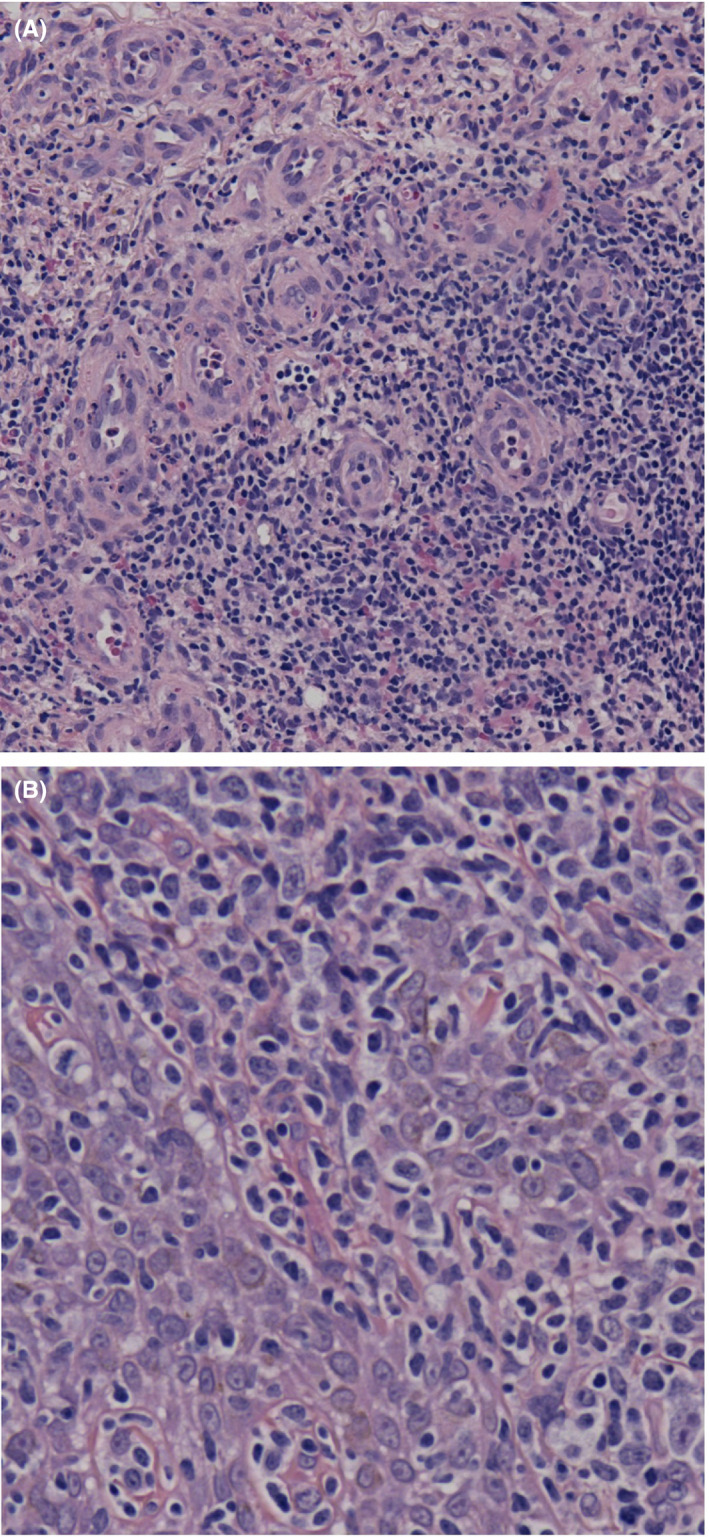
Skin biopsy showed atypical B cells in the dermis with large basophilic cytoplasm, irregular and vesicular chromatin (hematoxylin‐eosin‐saffron stain, A, original magnification ×20, B, original magnification ×40)

**Figure 3:**
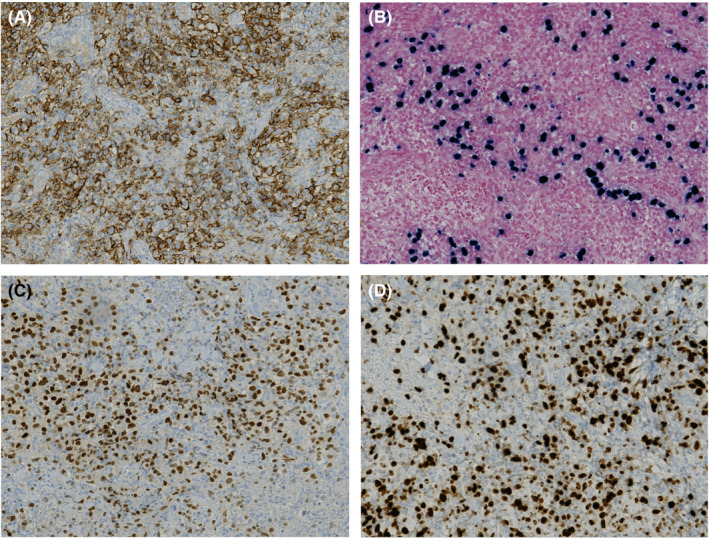
Immunohistochemical staining showed positivity for CD20 (A), EBER (B) and MUM‐1 (C) and a high Ki67 labelling index (D) (original magnification ×20)

**Figure 4:**
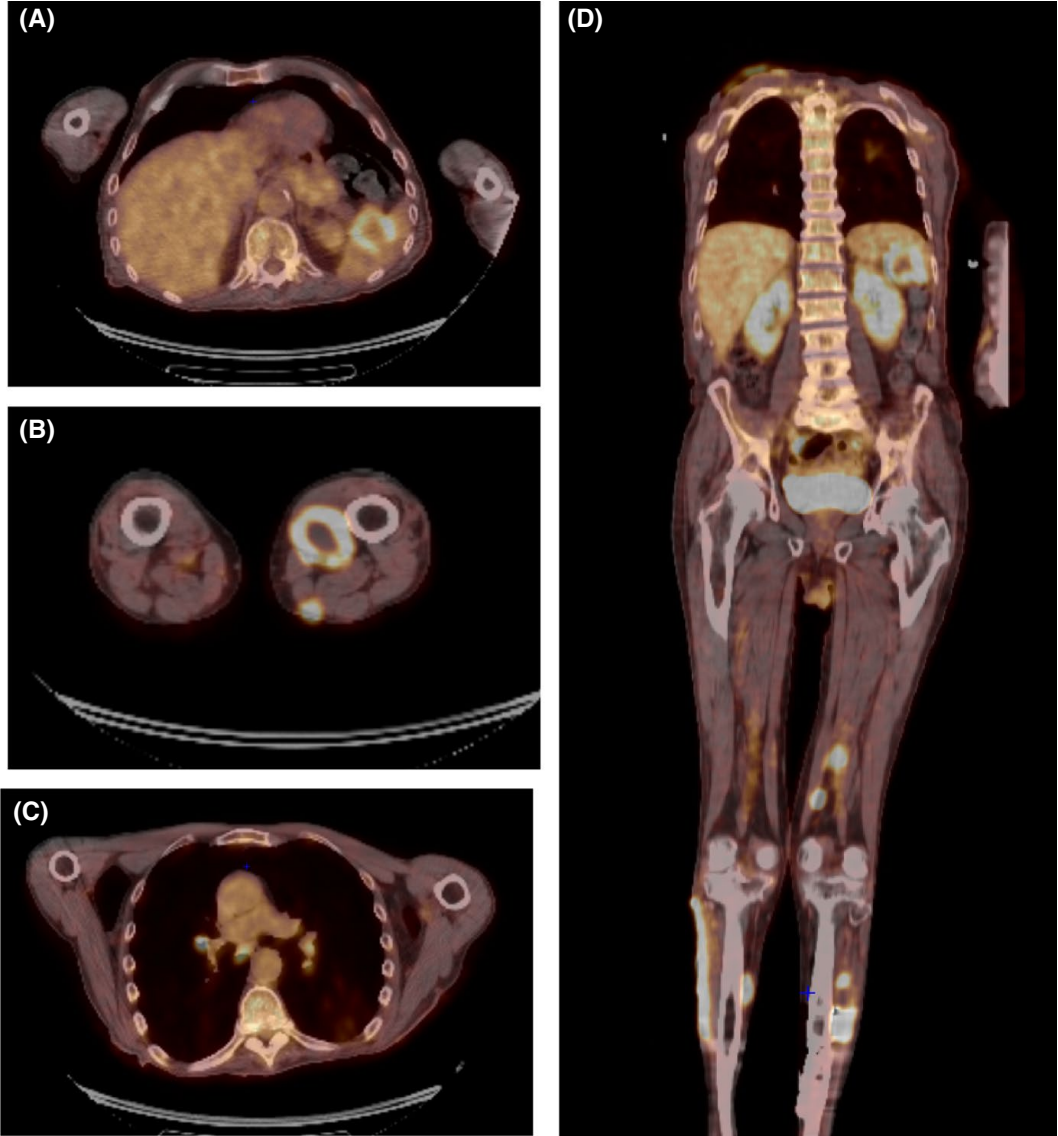
18F‐fluorodeoxyglucose positron‐emission tomography revealed hypermetabolic spleen (A) and bilateral hilar and mediastinal (B) involvement and several cutaneous and subcutaneous localizations (C: lower limbs, axial view; D: whole body, coronal view)

Skin biopsies showed a dense and deep infiltrate of neoplastic lymphoid cells with large basophilic cytoplasm, irregular and vesicular chromatin with oval to round small nucleoli (Figure 2). Most cells were huge, noncleaved, centroblasts‐like, and immunoblast‐like. Immunohistochemical staining showed a B‐cell population positive for CD20, CD30, PAX‐5, MUM‐1, and EBER (Figure 3A‐C), and negative for CD10 and BCL6 associated with a minority of CD2+, CD3+, CD5 + reactive T‐cell population. Mitotic figures were frequent with around 80% of tumor cells that were labelled with the Ki67 marker (Figure 3D). EBV viral load in blood was 8418 UI/mL. 18F‐fluorodeoxyglucose positron‐emission tomography showed spleen and mediastinum involvement and several cutaneous and subcutaneous localizations (Figure 4). Clonality testing on skin biopsies revealed monoallelic *IGH* rearrangement. Gene expression profile using reverse transcription‐multiplex ligation‐dependent probe amplification (RT‐MLPA)^4^ indicated an overexpression of EBER, Cyclin‐D1 and MYC but Cell of Origin (activated B‐cell/germinal center B‐cell) was unclassifiable. There was no evidence of bone marrow or peripheral blood involvement.

The diagnosis of primary cutaneous EBV‐positive DLBCL with rapid skin dissemination and systemic involvement was retained. Due to the patient's comorbidities, palliative therapy with corticosteroids and intravenous rituximab 375 mg/m^2^ for four weekly cycles was initiated. Unfortunately, skin lesions did not improve and the patient died four months after the diagnosis.

## 3 DISCUSSION

Viruses can lead to systemic lymphoma such as human T‐lymphotropic virus 1 (HTLV‐1) which causes T‐cell lymphoma^5^ or even more rarely human herpesvirus 8 (HHV8) associated with DLBCL.^6^ EBV is a ubiquitous lymphotropic herpesvirus that infects 80%‐90% of adults in the world.^7^ EBV is also highly associated with lymphoproliferative disorders such as Burkitt lymphoma,^8^ Hodgkin disease^9^ and extranodal natural killer (NK)/T‐cell lymphoma of nasal type.^10^

Based on the revised 4th Edition of the WHO classification of tumors of the hematopoietic system,^1^ EBV‐MCU; primary EBV‐positive DLBCL; plasmablastic lymphoma; lymphomatoid granulomatosis; cutaneous post‐transplant lymphoproliferatice disorders are EBV‐associated B‐cell neoplasia that can affect the skin. Among these entities, primary EBV‐DLBCL must be distinguished from EBV‐MCU that may commonly affect the elderly. Indeed, EBV‐MCU typically affects immunosuppressed patients and presents as a solitary ulceration in the skin, oral cavity, or gastrointestinal tract without systemic involvement.^3^ This pseudomalignant entity has a good prognosis with spontaneous regression of ulcerations in 25% of patients.^11^

Primary cutaneous EBV‐positive DLBCL is an exceptional entity which seems to be more frequent in Asian populations than in Western countries with a slight male predominance.^2^ Frequency of primary cutaneous EBV‐positive DLBCL range from 8.7% to 11.4% in Asian countries, as compared to less than 5% in Western countries.

This rare entity predominantly affects the immunosenescent elderly and is characterized by an unfavorable prognosis and poor response to treatment.^2^ The age‐related immunodepression, so called immunosenescence, modifies T‐cell homeostasis through a decrease of thymic output of naïve T cells due to thymic involution and an accumulation of viral specific CD8 + T cells^12^ associated with profound functional changes in CD4 + T cells,^13^ facilitating the growth of a state of chronic inflammatory process called “inflammaging” probably involved in EBV‐reactivation and neoplasms onset. However, the molecular details of EBV reactivation process remains largely unclear. The exanthem of mononucleosis (more frequently observed after administration of ampicillin), oral hairy leukoplasia in immunosuppressed HIV‐positive and HIV‐negative patients, papular acrodermatitis of childhood (Gianotti‐Crosti syndrome) and acute genital ulcers (ulcer of Lipschutz) are the most common mucocutaneous manifestations related to EBV infection.^14^, ^15^ Other cutaneous manifestations have been linked to EBV such as hypersensitivity of mosquito bites, hydroa vacciniforme, and drug reaction with eosinophilia and systemic symptoms.^15^

The clinical presentations of primary cutaneous EBV‐positive DLBCL are variable, ranging from a single nodule to multiple plaques.^16^ An involvement of other organs should be investigated such as bone marrow, lungs, gastrointestinal tract, and ear, nose and throat (ENT) region.^17^ Primary cutaneous EBV‐positive DLBCL is diagnosed with a skin biopsy with or without an excisional biopsy of a suspicious lymph node, which always shows positivity for EBER through in situ hybridization analysis.^18^ Typically, EBV‐DLBCL has a nongerminal center (non‐GC) phenotype (CD10‐, BCL‐6 ±, MUM1+) with an expression of pan B‐cell markers by immunohistochemical staining (CD19, CD20, CD22, CD79a, PAX‐5).

When possible, the first‐line treatment may include rituximab monotherapy as single agent^19^ or associated with anthracycline‐based chemotherapy such as CHOP (cyclophosphamide, doxorubicin, vincristine, prednisone) by analogy with post‐transplant lymphoproliferative disorders and non‐EBV‐DLBCL treatments^20^, ^21^ because no standard of care exist in this very rare disease. In addition, the treatment is often challenging because of the severity of the pathology and patients’ age and comorbidities. EBV viral load, as measured by quantitative molecular analysis of the viral genome in blood, may serve as a biomarker for predicting and monitoring the course of EBV‐associated diseases.^22^

## 4 CONCLUSION

To conclude, we report here an exceptional cause of multiple cutaneous ulcers in an elderly patient corresponding to a primary cutaneous EBV‐positive DLBCL with a very uncommon presentation and a dismal clinical course. This case highlights this very rare immunosenescence‐associated lymphoid malignancy as an edifying cause of multiple skin ulcerations, to be distinct from other leg ulcers unrelated to arterial and venous disease. Although rare, this etiology must be mentioned in case of cutaneous ulcers in elderly patients with impaired general condition and justifies performing early skin biopsies. The treatment remains to be challenging and there is a need for alternative therapeutic approach because of the dismal clinical course and patients’ age and comorbidities.

## CONFLICT OF INTEREST

None declared.

## AUTHOR CONTRIBUTIONS

BT and VC: wrote the manuscript. SD, HT, and FJ and PJ: reviewed the manuscript. All authors were involved in the care of the patient. All authors read and approved the final manuscript.

## ETHICAL APPROVAL

Patient's written informed consent to publication was obtained.

## References

[1] Swerdlow SH , Campo E , Pileri SA , et al. The 2016 revision of the World Health Organization classification of lymphoid neoplasms. Blood. 2016;127(20):2375‐2390. 26980727 10.1182/blood-2016-01-643569 PMC4874220

[2] Ok CY , Papathomas TG , Medeiros LJ , Young KH . EBV‐positive diffuse large B‐cell lymphoma of the elderly. Blood. 2013;122(3):328‐340. 23649469 10.1182/blood-2013-03-489708 PMC3779382

[3] Ikeda T , Gion Y , Yoshino T , Sato Y . A review of EBV‐positive mucocutaneous ulcers focusing on clinical and pathological aspects. J Clin Exp Hematop. 2019;59(2):64‐71. 31257347 10.3960/jslrt.18039 PMC6661964

[4] Mareschal S , Ruminy P , Bagacean C , et al. Accurate Classification of Germinal Center B‐Cell–Like/Activated B‐Cell–Like Diffuse Large B‐Cell Lymphoma Using a Simple and Rapid Reverse Transcriptase‐Multiplex Ligation‐Dependent Probe Amplification Assay: A CALYM Study. J Mol Diagn. 2015;17(3):273‐283. 10.1016/j.jmoldx.2015.01.007 25891505

[5] Kogure Y , Kataoka K . Genetic alterations in adult T‐cell leukemia/lymphoma. Cancer Sci. 2017;108(9):1719‐1725. 28627735 10.1111/cas.13303 PMC5581529

[6] Nador RG , Cesarman E , Chadburn A , et al. Primary effusion lymphoma: a distinct clinicopathologic entity associated with the Kaposi’s sarcoma‐associated herpes virus. Blood. 1996;88(2):645‐656. 8695812

[7] Tzellos S , Farrell PJ . Epstein‐barr virus sequence variation‐biology and disease. Pathogens. 2012;1(2):156‐174. 25436768 10.3390/pathogens1020156 PMC4235690

[8] De‐Thé G . The epidemiology of Burkitt’s lymphoma: evidence for a causal association with Epstein‐Barr virus. Epidemiol Rev. 1979;1:32‐54. 232877 10.1093/oxfordjournals.epirev.a036213

[9] Anagnostopoulos I , Herbst H , Niedobitek G , Stein H . Demonstration of monoclonal EBV genomes in Hodgkin’s disease and Ki‐1‐positive anaplastic large cell lymphoma by combined Southern blot and in situ hybridization. Blood. 1989;74(2):810‐816. 2546633

[10] Kimura H , Ito Y , Kawabe S , et al. EBV‐associated T/NK‐cell lymphoproliferative diseases in nonimmunocompromised hosts: prospective analysis of 108 cases. Blood. 2012;119(3):673‐686. 22096243 10.1182/blood-2011-10-381921

[11] Dojcinov SD , Venkataraman G , Raffeld M , et al. EBV positive mucocutaneous ulcer–a study of 26 cases associated with various sources of immunosuppression. Am J Surg Pathol. 2010;34:405‐417. 20154586 10.1097/PAS.0b013e3181cf8622 PMC6437677

[12] Franceschi C , Bonafè M , Valensin S . Human immunosenescence: the prevailing of innate immunity, the failing of clonotypic immunity, and the filling of immunological space. Vaccine. 2000;18(16):1717‐1720. 10689155 10.1016/s0264-410x(99)00513-7

[13] Fülöp T , Dupuis G , Witkowski JM , Larbi A . The role of Immunosenescence in the development of age‐related diseases. Rev Investig Clin. 2016;68(2):84‐91. 27103044

[14] Lernia VD , Mansouri Y . Epstein‐Barr virus and skin manifestations in childhood. Int J Dermatol. 2013;52(10):1177‐1184. 24073903 10.1111/j.1365-4632.2012.05855.x

[15] Hall LD , Eminger LA , Hesterman KS , Heymann WR . Epstein‐Barr virus: dermatologic associations and implications: part I. Mucocutaneous manifestations of Epstein‐Barr virus and nonmalignant disorders. J Am Acad Dermatol. 2015;72(1):1‐19; quiz 19–20. 25497917 10.1016/j.jaad.2014.07.034

[16] Eminger LA , Hall LD , Hesterman KS , et al. Epstein‐Barr virus: dermatologic associations and implications. J Am Acad Dermatol. 2015;72:21‐34. 25497918 10.1016/j.jaad.2014.07.035

[17] Oyama T , Yamamoto K , Asano N , et al. Age‐related EBV‐associated B‐cell lymphoproliferative disorders constitute a distinct clinicopathologic group: a study of 96 patients. Clin Cancer Res. 2007;13:5124‐5132. 17785567 10.1158/1078-0432.CCR-06-2823

[18] Beltran BE , Castro D , Paredes S , Miranda RN , Castillo JJ . EBV‐positive diffuse large B‐cell lymphoma, not otherwise specified: 2020 update on diagnosis, risk‐stratification and management. Am J Hematol. 2020. [Epub ahead of print]. 10.1002/ajh.25760 32072672

[19] Styczynski J , van der Velden W , Fox CP , et al. Management of Epstein‐Barr Virus infections and post‐transplant lymphoproliferative disorders in patients after allogeneic hematopoietic stem cell transplantation: Sixth European Conference on Infections in Leukemia (ECIL‐6) guidelines. Haematologica. 2016;101(7):803‐811. 27365460 10.3324/haematol.2016.144428 PMC5004459

[20] Park S , Lee J , Ko YH , et al. The impact of Epstein‐Barr virus status on clinical outcome in diffuse large B‐cell lymphoma. Blood. 2007;110(3):972‐978. 17400912 10.1182/blood-2007-01-067769

[21] Feugier P , Van Hoof A , Sebban C , et al. Long‐term results of the R‐CHOP study in the treatment of elderly patients with diffuse large B‐cell lymphoma: a study by the Groupe d'Etude des Lymphomes de l'Adulte. J Clin Oncol. 2005;23(18):4117‐4126. 15867204 10.1200/JCO.2005.09.131

[22] Kimura H , Kwong Y‐L . EBV viral loads in diagnosis, monitoring, and response assessment. Front Oncol. 2019;9:62 10.3389/fonc.2019.00062 30809508 PMC6379266

